# Evidence for mutual assessment in a wild primate

**DOI:** 10.1038/s41598-017-02903-w

**Published:** 2017-06-07

**Authors:** Marcela E. Benítez, David J. Pappano, Jacinta C. Beehner, Thore J. Bergman

**Affiliations:** 1 Department of Psychology, Language Research Center, Georgia State University, Atlanta, Georgia USA; 2 Department of Ecology and Evolutionary Biology, Princeton University, Princeton, New Jersey USA; 3 Department of Anthropology, University of Michigan, Ann Arbor, Michigan USA; 4 Department of Psychology, University of Michigan, Ann Arbor, Michigan USA; 5 Department of Ecology and Evolutionary Biology, University of Michigan, Ann Arbor, Michigan USA

## Abstract

In aggressive interactions, game theory predicts that animals should assess an opponent’s condition relative to their own prior to escalation or retreat. Despite the benefits of such *mutual assessment*, few studies have been able to reject simpler assessment strategies. Here we report evidence for mutual assessment in a wild primate. Gelada (*Theropithecus gelada*) males have conspicuous loud calls that may function as a signal of male quality. “Leader” males with harems putatively use loud calls to deter challenges from non-reproductive “bachelor” males. By contrast, leader males pose no threat to each other and congregate in large groups for a dilution effect against bachelors. In playback experiments and natural observations, gelada males responded to loud calls according to both their own *and* their opponent’s attributes. Although primates routinely classify others relative to themselves using individual attributes, this represents some of the first direct evidence for mutual assessment in primate signaling contests.

## Introduction

Limited resources lead animals into contests. Because aggressive contests are costly, game theory predicts that contestants will assess the costs and benefits of a particular contest before escalating^1, 2^. Contestants with a high ability to compete (i.e., high resource holding potential – RHP) should escalate the contest, while those with a low ability to compete (low RHP) should withdraw. Despite the simplicity of this prediction, there is enormous debate about how animals make these decisions^3^. It stands to reason that a contestant should gather information about their opponent’s condition and compare that to their own (*mutual assessment* ^2^). However, many empirical studies find it difficult to reject “simpler” assessment strategies^4^ such as *self-assessment* (relying solely on one’s own condition^5^), or *opponent-only assessment* (relying solely on a rival’s condition^6^). For example, when an inferior contestant withdraws from an aggressive contest with an opponent, the contestant may indeed be using mutual assessment, or they may simply be withdrawing because the damage incurred was too high. Therefore, a “cumulative” *self-assessment* strategy is difficult to distinguish from a “sequential” *mutual assessment* one^4^.

Non-contact displays – such as those involving animal signals – avoid this problem entirely^7^. Contestants do not accumulate sufficient costs during displays for a cumulative assessment strategy to operate. Therefore, measuring receiver responses based on the relative quality of the signaler and the receiver makes for a strong test of mutual assessment. However, within the vast literature documenting receiver responses to putative signals based on the quality of the signaler^8–19^, only a handful of studies also examined receiver responses based on the relative quality of the signaler *and the receiver* ^7, 19–22^. Thus, the current evidence for many signals is only sufficient for identifying opponent-only assessment. In practice, however, it is likely that many of these taxa may be using mutual assessment.

In non-human primates (hereafter, “primates”), there is very little evidence for mutual assessment based solely on the content encoded in a signal for two reasons. First, experimental manipulations are necessary. In one of the few captive experiments that sought to examine mutual assessment (e.g., manipulated scrotal color and staged contests in male vervet monkeys, *Chlorocebus pygerythrus*), the results were inconclusive possibly because it is near impossible to recreate realistic scenarios that males typically encounter in the wild^22^. Second, primates tend to rely predominantly on social information that derives from individual recognition (not signals) to guide their interactions^23^. The use of this social information can be quite sophisticated (e.g., eavesdropping)^24^ or quite simple (e.g., requiring nothing more than recognizing an individual)^23^. For example, in primate systems with linear dominance hierarchies, the dominance rank of an individual determines the outcome of most social interactions^25–27^. Typically, these interactions are not thought to be based on the relative Resource Holding Potential (RHP) of contestants at the time of the interaction, but rather based on social knowledge of contestants derived from a recent history of interactions with them^28^. However, results from previous research on mutual assessment have not been able to entirely distinguish whether primate subjects use *individual identity* or *quality signals* as the underlying basis for assessment, even in cases where playback experiments were able to manipulate the individual and/or the signal.

For example, playback experiments in chacma baboons (*Papio ursinus*) have shown that subjects respond to loud call displays based on the relative RHP of contestants^19, 25, 26^. Specifically, based on simulated loud call contests, chacma males were more likely to enter vocal contests if the opponent’s rank was similar to their own^26^, they were able to discriminate between the relative ranks of “third party” contestants^25^, and they were more interested in calls that were manipulated to suggest a higher quality male^19^. Combined, these three studies suggest that mutual assessment may be occurring in chacma baboons. However, the first two studies^25, 26^ and half of the third study^19^ were conducted entirely with familiar rivals. In a parallel study on chacma male grunts (soft vocalizations that are unlikely to carry information about the sender’s competitive ability), researchers also observed that the strongest responses occurred between similarly-ranked males^29^. Therefore, because known males were used, it is impossible to distinguish whether chacma male assessment hinged on individual recognition (knowing who is calling) or signal strength (knowing the quality of the caller). In the only experiment to use unknown callers, the subjects did not alter their responses according to their own rank – they all attended more strongly to the higher quality loud call^19^. Therefore, although these results are certainly suggestive, they fall just short of providing clear evidence of mutual assessment in the context of animal signals. Here we examined whether a close relative to chacma baboons, the gelada (*Theropithecus gelada*), uses mutual assessment when hearing loud calls from other males.

Geladas present an unusually tractable system for experimentally studying assessment in primates. Geladas have a vocal signal that is used in male-male competition, allowing us to use playback experiments to disentangle various assessment strategies. Geladas’ reliance on vocal signals likely relates to their large, fluid social systems where, in contrast to closely-related species (including chacma baboons), they frequently interact with unfamiliar individuals^30^. Geladas are large-bodied, terrestrial primates that live in the high-montane grasslands of Ethiopia^31^. They congregate in a large, fluid, multi-level society composed primarily of harems (“reproductive units” – hereafter “unit”) comprising one harem-holding male (“leader male”), 1–12 related adult females and their offspring, and occasionally one or more subordinate males (“follower males”). Leader males (often joined by follower males) fiercely guard their harems from “bachelor males” that reside in all-male groups at the periphery of the larger aggregations of units^32^. Importantly, bachelor males gain reproductive access to females primarily by challenging and defeating a leader male^33^. By contrast, leader males pose no threat to each other^33^ and frequently gather into large foraging aggregations^34^ for a putative “dilution effect” against predators^35^ and/or bachelors^36^.

Leader males deter bachelors from challenging them by engaging in ritualized vocal displays that culminate in a series of loud calls^32^. These displays begin when a leader male approaches, threatens, and solicits a chase from a group of bachelor males^32^. The display itself does not immediately result in aggression between the leader and the bachelor males, but is thought instead to transmit information on the strength and/or condition of the initiating leader male to the recipient bachelor males^33, 37^. The end of each display is punctuated with one or more bouts of loud calls by the leader male. While only one leader male is chased at a time, these displays elicit the attention from other males, and the loud calls themselves appear to be “contagious”; that is, after each display, between 2–13 leader males (and occasionally follower males as well) produce additional loud calls of their own^33^. Additionally, each display is often followed by subsequent displays from other leader males, with each male taking a turn (i.e., soliciting a chase and ending with a bout of loud calls), venturing away from his harem to engage with the bachelors and produce loud calls before returning to his females^33^. Bachelors do not produce loud calls during these displays^33^.

Previous research in geladas reported that leader males that display more frequently were less likely to be targeted by bachelors, suggesting that the *quantity* of these displays serves to deter rivals^33^. But, in addition to the quantity of loud calls produced, recent evidence also suggests that the *quality* of these loud calls is important for rival assessment^37^. Specifically, the males with the highest RHP in gelada society (e.g., prime-aged, high-status males) utter the most calls per bout, produce calls that are the lowest in overall frequency measures, and exhibit the greatest vocal range^37^. Thus, the loud calls themselves appear to be honest signals of male RHP, and bachelor males could use RHP information encoded in these calls for identifying relatively low-quality males (e.g., old males, low-status males, exhausted males)^37^.

By contrast, leader males do not assess bachelors. Leader males are always on defense, never offence, from bachelor males. However, leader males do have the potential to assess *other leader males* via these calls^37^. If indeed leader males rely on a putative dilution effect to avoid being challenged by bachelor males, then each leader’s position is secure only if they, themselves, have a higher RHP than the other leader males around them. Thus, leader males can use RHP information encoded in these calls for identifying situations when they are surrounded by relatively strong males (and are, thus, weaker by comparison).

**Figure 1:**
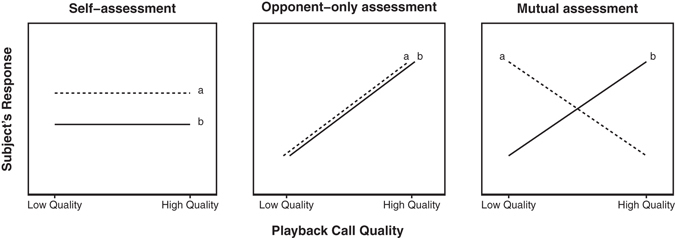
Predictions for bachelor male (**a**) and leader male (**b**) responses to low- and high-quality simulated loud calls for three assessment strategies: self-assessment, opponent-only assessment, and mutual assessment.

We used a playback experiment as well as observations of natural behavior in wild geladas to investigate the rival assessment strategy used by males. We examined male responses to both experimental and natural loud calls of varying quality. If gelada males rely only on *self-assessment* in male contests, we predicted that neither leaders nor bachelors would respond differently to low- and high-quality calls (Fig. 1a). If gelada males rely on *opponent-only assessment*, we predicted that all subjects would attend more strongly to high-quality calls than low-quality calls regardless of their own status (Fig. 1b; note that the direction of this response could also be reversed). However, if gelada males rely on *mutual assessment* in male contests (Fig. 1c), we predicted that male subjects would respond to loud calls based on the combined information about themselves (i.e., their own status and/or RHP) and the quality of their rival (i.e., call quality). Specifically, we expected: (1) **bachelor males** to attend more to low-quality calls (a weak rival) because this represents a prime opportunity for a takeover; (2) **leader males** to attend more to high quality calls (a situation that weakens their relative security in the group, and one that indicates they might soon be challenged) – (3) particularly if they themselves have high RHP; (4) **high RHP leader males** to not just attend to high quality calls, but to advertise their own quality by subsequently participating in the display; and (5) **females** to not discriminate between call quality (because loud calls are used in male-male competition rather than female choice).

## Results

### Do males attend differently to high- and low-quality calls based on their own status?

We conducted a playback experiment on 60 adult geladas (20 females, 20 leader males, and 20 bachelor males) using previously recorded loud calls obtained during naturally-occurring displays between adult males (7 high-quality bouts and 7 low-quality bouts were used to construct 10 playback sets each containing a unique combination of one high- and one low-quality loud call bout from different males). Each subject heard both a high-quality loud call (one caller) and a low-quality loud call (a different caller). We visually recorded each subject’s response to each call type (randomized for order of presentation) and examined six response variables (*look duration, approach duration, latency to look, latency to approach, approach distance*, and *time to resume activity*), which were reduced using factor analysis. The factor analysis resulted in two latent factors, (1) an “approach” response, and (2) a “look” response, with Eigenvalues >1, together explaining 90.68% of the total variance. Factor 1 (“approach response”) accounted for 64.18% of the variance and loaded heavily on *approach duration*, *approach distance*, and *latency to approach*. Factor 2 (“look response”) accounted for 26.50% of the variance and loaded heavily on *look duration*, *latency to look*, and *time to resume activity* (Table 1).

**Table 1:** Loadings from Factor Analysis.

*Variables*	Factor 1	Factor 2
	*Approach response*	*Look response*
Look duration (s)	0.16	**0.92**
Look latency (s)	−0.07	**−0.92**
Move duration (s)	**0.97**	0.17
Move latency (s)	**−0.95**	−0.19
Distance moved* (m)	**0.93**	0.20
Resume activity (s)	**0.45**	**0.84**
Eigenvalue	3.85	1.59
Variance	64.18%	26.50%

*Distance moved towards speaker (*not away*).

To examine whether social status and/or call quality (high or low) determined a subject’s response, we constructed two Linear Mixed Models (LMMs) with each factor score as the dependent variable. In each model, we included social status (leader, bachelor, or female), call quality (high or low), and an interaction between them as predictors and controlled for call order (fixed) and subject (random). To further assess if bachelors and leaders differentiated between call-quality, we conducted additional pairwise contrasts and adjusted the p-value accordingly.

**Figure 2:**
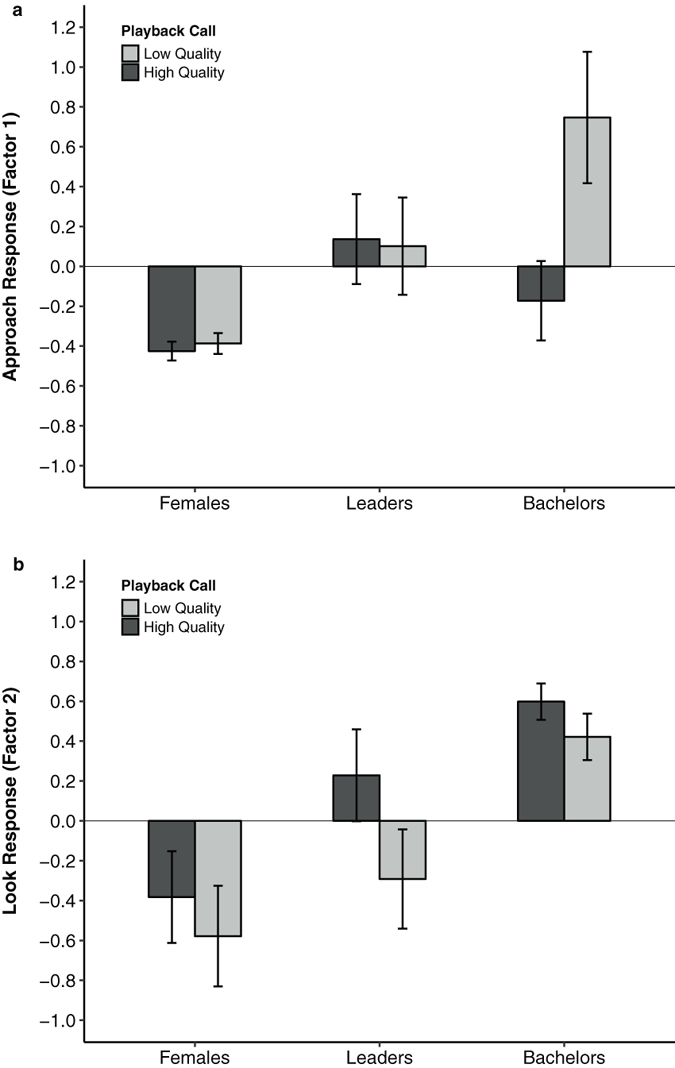
Subject responses (Mean of factor scores + SEM) to simulated high and low quality loud calls from bachelors, leaders, and females. (**a**) Factor 1 is a composite score where larger values indicate a stronger “approach” response. (**b**) Factor 2 is a composite score where larger values indicate a stronger “look” response. See text for details.

For Factor 1 (“approach response”), we found a significant interaction between bachelors and call quality (β = 0.25, s.e. = 0.11, t = 2.202, *p* = 0.032; Table 2). Bachelors approached low-quality calls significantly more than females and leaders (Fig. 2a). Bachelors were also more likely to approach low-quality calls than high-quality calls (β = −0.27, s.e. = 0.08, t = −3.353, *p* = 0.014; Table 2), but neither leaders (β = 0.02, s.e. = 0.08, t = 0.199, *p* = 0.843) nor females (β = 0.02, s.e. = 0.08, t = 0.234, *p* = 0.816) differed in whether they approached either call type (Table 2). In fact, females rarely approached the speaker (Table S1).

**Table 2:** Results from LMMs.

*Predictors*	Approach Response (Factor 1)				Look Response (Factor 2)			
	*Beta*	*Std. Err*	*t*	*p*	*Beta*	*Std. Err*	*t*	*p*
(Intercept)	−0.29	0.07	−3.954	<0.001	−0.36	0.21	−1.684	0.096
Status (Bachelor)	−0.03	0.10	−0.291	0.771	0.99	0.29	3.389	**0.001**
Status (Leader)	0.17	0.10	1.661	0.100	0.61	0.29	2.110	**0.038**
Call Quality (Low)	0.02	0.08	0.234	0.816	−0.19	0.18	−1.042	0.302
Call Order (First)	0.05	0.05	1.145	0.257	−0.06	0.11	−0.568	0.572
Bachelor × Low Quality	0.25	0.11	2.202	**0.032**	0.01	0.26	0.029	0.977
Leader × Low Quality	−0.03	0.11	−0.306	0.761	−0.33	0.26	−1.280	0.206
*full vs. null model*	χ^2^ = 15.64, p = 0.016				χ^2^ = 23.94, p < 0.001			
*Contrasts*								
Bachelor: High – Low	−0.27	0.08	−3.353	0.014*	0.18	0.18	1.000	0.321
Leader: High - Low	0.02	0.08	0.199	0.843	0.52	0.18	2.856	0.006*

^*^Bonferroni adjusted alpha level of <0.025.

By contrast, leaders were more likely to look (Factor 2) towards high-quality calls than low-quality calls (β = 0.52, s.e. = 0.18, t = 2.856, *p* = 0.006; Fig. 2b). Yet, neither bachelors (β = 0.18, s.e. = 0.18, t = 1.000, *p* = 0.321) nor females (β = −0.19, s.e. = 0.18, t = −1.042, *p* = 0.302) distinguished between call type in terms of looking time (Table 2). In general, both bachelors (β = 0.99, s.e. = 0.29, t = 3.389, *p* = 0.001) and leader males (β = 0.61, s.e. = 0.29, t = 2.110, *p* = 0.038) spent more time looking towards the speaker than females did. We found no effect of call order in either model (Factor 1; β = 0.05, s.e. = 0.05, t = 1.145, *p* = 0.257: Factor 2; β = −0.06, s.e. = 0.11, t = −0.568, *p* = 0.572).

**Figure 3:**
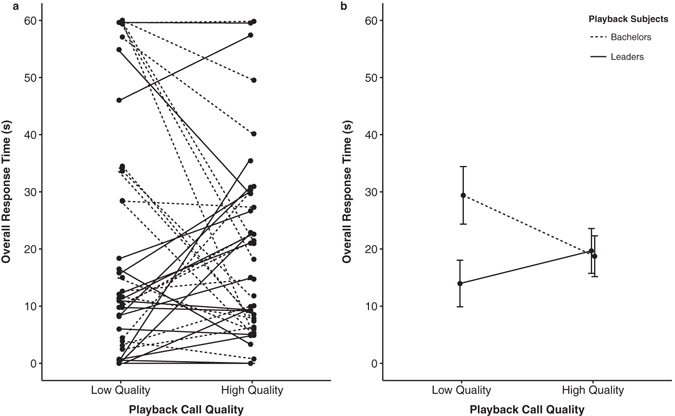
Status difference for males in overall response time to high- and low-quality calls. Figure 3 represents both (**a**) within subject differences for 20 bachelors and 20 leaders, and (**b**) mean total response time (+SEM) to different call types.

In addition to the Factor Analysis, we further examined overall response time, a measure of how long each individual spent investigating the source of the call (*look duration* + *move duration*), to assess whether status or call type affected the overall strength of a male’s response. Supporting the previous results, we found a significant effect of call quality for bachelors and a significant interaction between social status and call quality. Bachelors spent more time oriented towards loud call bouts of low-quality (low quality; β = 10.66, s.e. = 3.82, t = 2.787, *p* = 0.008; Fig. 3a,b) while leader males spent more time oriented towards loud call bouts of high-quality (leader x low-quality; β = −16.35, s.e. = 5.41, t = −3.024, *p* = 0.004; Fig. 3a,b). In general, bachelors and leaders did not differ in their overall total orientation time to high-quality loud calls (β = 0.93, s.e. = 5.92, t = 0.157, *p* = 0.876; Fig. 3).

### Do males attend differently to high- and low-quality calls based on their own quality?

Males’ responses to differences in call quality were based on their own categorical differences in status as a leader or bachelor. We additionally wanted to examine whether males further differentiated playback stimuli based on their own “quality” (i.e., using the quality of their own loud calls as a proxy for overall “quality”^37^). The sample for this analysis (N = 11) was only a subset of the leader males used for the first analysis (we did not have recordings from all subjects, and generally only leader males produce loud calls^33^). Note that because low-quality males rarely produce loud calls, the leader males included in this analysis disproportionately comprise males whose loud calls are mid- to high-quality. We predicted that the previous result was due mainly to the high-quality leader males responding to the high-quality call type. We established a *call quality score* for each subject’s loud calls in the same way that we determined high- and low-quality calls for the playback experiment.

**Figure 4:**
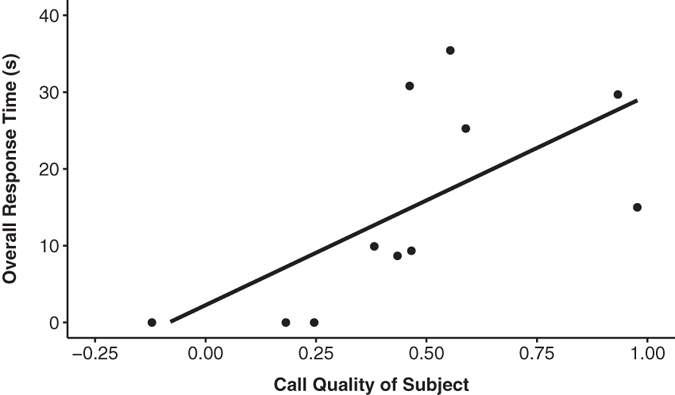
Overall response time (s) to the high-quality playback call in relation to the subjects own call quality.

For each call type (low, high), we compared each subject’s overall response time to his own call quality score. In response to the simulated low-quality calls, we found no relationship between the subject’s call quality score and his overall response time (r_s_ = 0.489, p = 0.127). However, in response to the simulated high-quality calls, leader males with high call quality scores themselves, responded more strongly than those with low quality scores (r_s_ = 0.752, p = 0.008; Fig. 4).

### Are males more likely to join a loud call display when they hear calls of similar quality to their own?

The previous two results suggest that we can predict a male’s response to the quality of a loud call by using experimental stimuli – bachelor males responded strongly to simulated low-quality calls and leader males responded strongly to simulated high-quality calls. Within leader males, high-quality leaders responded strongest to high-quality calls suggesting that leaders attend to both the quality of the caller and their own quality. Next, we wanted to determine if these same results hold true in natural observations of male contests. Loud call displays often serve as a catalyst for other unit males to join in with loud calls of their own. We predicted that high-quality leader males will be more likely to enter a loud call display when the display includes other males of high quality. To test these predictions, we used behavioral observations and loud call recordings from 20 unit males (16 leader males, 4 follower males) across 291 loud call displays, recording 423 loud calls from all 20 males.

We then examined whether male A (subject) was more likely to participate in a display given that male B also displayed (binomial distribution). We included relative call quality (the difference between the call quality scores of both males), caller “familiarity” (using social network analysis), and leader/follower status in the models as fixed effects; and we included the identification of both males as random effects.

Males were more likely to display with males of similar call quality scores to their own (β = −0.62, s.e. = 0.31, z = −2.042, *p* = 0.041; Table 3), with caller familiarity having little effect (β = 0.25, s.e. = 0.14, z = 1.808, *p* = 0.071; Table 3).

**Table 3:** Results from General Linear Mixed Model (GLMM).

Predictors	Beta	Std. Err	z	p
(Intercept)	−2.78	0.45	−6.132	8.69 e-10
Difference in call quality	−0.62	0.31	−2.042	**0.041**
Same group (yes)	0.25	0.14	1.808	0.071
*full vs. null model*	χ^2^ = 7.05, p = 0.029			

## Discussion

In simulated signal displays, gelada males, but not females, discriminated between loud calls based on the acoustic quality of the signal as well as their own status and quality. Specifically, bachelor males – males that must compete to gain reproductive access to females – exhibited a stronger response to low-quality loud calls, while leader males exhibited a stronger response to high-quality loud calls. Furthermore, within leader males from whom we had loud calls (a sample biased towards mid- to high-RHP males), we found that higher-RHP males themselves (based on their call quality) responded more strongly to the high-quality stimuli than did lower-quality males. Finally, in natural observations, leader males were more likely to join loud call displays when their own calls were of similar quality to the other males involved in the display. In all three cases, a male’s response to other males’ loud calls was based on both their own RHP and that of the caller (coded into the quality of the signal). Taken together, these findings support the hypothesis that gelada males use a mutual assessment strategy, rather than a self- or opponent-based one. These data provide some of the first evidence for a mutual assessment strategy using signals for a non-human primate.

Although bachelor males attended to both high- and low-quality loud call bouts (Fig. 2b), they only approached the hidden speaker (“escalated”) when they were played the low-quality call (Fig. 2a). In playback studies, approach behaviors (e.g., approach distance, approach rate, latency to approach) represent more “intense” measures of interest in the signal than looking time alone^38–41^. This is especially true in the study of aggressive signals where approaching the source of the call is a relatively high-cost response, as it implies an interest in engaging the caller^42, 43^. In support of this, males that approached the speaker reached (or passed) the source of the call (mean approach distance 32.7 m, mean speaker distance 28.9 m), and 73% of these approaches were accompanied with visual and vocal threats. When confronted with a potentially weak rival, bachelors may benefit from an escalated response (i.e., an approach) because successful challenges can result in reproductive access to females^33, 44, 45^. By contrast, when confronted with a potentially strong rival, bachelors may suffer severe (and possibly fatal) costs from an escalated response^32^. Our results suggest that bachelor males assess the quality of leader males by attending to these loud calls, and they use information gleaned from these calls to make decisions about which males to challenge and which to avoid.

By contrast, leader males rarely approached the speaker regardless of call quality (Fig. 2a). A strong approach is especially risky for a leader male as it requires him to leave his female unattended with bachelor males in close proximity. However, leader males did spend more time attending to the high-quality calls compared to the low-quality ones (Fig. 2b), with the strongest responses deriving from the leader males exhibiting the highest-RHP (as measured by their own loud call quality, Fig. 4). The motivation for leader males to attend to (and, engage in) call displays presumably derives from the need to showcase their own quality in the midst of bachelors. The large aggregations of geladas (sometimes numbering over 1200 individuals) have been hypothesized to create a “dilution effect” against predators^35^, but also against bachelors^36^. Indeed, at least one feature of loud call displays (how often a leader male participated in displays^33^) was negatively associated with his likelihood of takeover. Therefore, leader males should broadcast their loud calls when they “compare well” to displaying males around them. In support of this, leader males were more likely to participate in natural loud call displays when their call quality was similar to the males calling around them.

Similar results have been reported for chacma baboons (*Papio ursinus*) where males were more likely to loud call with males of similar dominance rank^25^. However, the male baboons were likely using social knowledge (monitoring other males’ ranks and attending to acoustic cues of identity) to assess one another, not signals^19, 25^. Indeed, mutual assessment using social knowledge appears routine among primates. In primate societies, interactions are structured by dyadic properties such as relative rank or kinship that require animals to account for the behavior of other individuals *in relation to themselves*. The novelty of our finding is in demonstrating that primates can use signals to perform mutual assessments while interacting with completely unfamiliar individuals. This use of signals to guide interactions is particularly useful in geladas where their large groups and fission-fusion social system^46^ require males to consistently monitor the quality of *unfamiliar* males.

Importantly, bachelors, by successfully defeating the resident harem-holding male, become leaders. As males transition from bachelors to leaders, the information an individual pays attention to is likely to change with this change in status. Other primate species have been shown to monitor changes in other individuals’ dominance ranks and social relationships over time^47^. In chacma baboons, for example, males track temporary changes in the status of other males’ consortship but, once again, the results of this study were likely based on identity information, not signals^48^. In the case of geladas, information acquired from quality signals may be the only way to successfully navigate such large social groups of unknown conspecifics.

Unlike males, gelada females did not differentiate between high- and low- quality calls. Indeed, they rarely attended to either call (Fig. 2a,b). There has been considerable debate as to whether loud calls in primates evolved to attract mates or to deter competitors^49, 50^. One of the strengths of this study is that both females and males were tested within the same design. Our results indicate that gelada loud calls evolved as a signal for assessing rivals and not attracting mates.

One promising avenue for future research will be to assess how group dynamics influence assessment strategies in social animals in a natural context^23^. For example, if the composition of social groups is dynamic, we might expect males to rely on information gleaned from signals rather than individual recognition and social knowledge when assessing rivals. More studies that combine experiments with natural observations of assessment behavior are necessary to understand the role of assessment strategies in social animals.

## Methods

### Study site and subjects

Research was conducted on a population of wild geladas living in the Simien Mountains National Park, Ethiopia from Feb-Dec 2013. The University of Michigan Gelada Research Project has been collecting long-term behavioral and demographic data on this population since January 2006. All males were individually recognizable and habituated to observers on foot (approach distance <3 m). Methods include a combination of playback experiments and behavioral observations. We have adhered to the Guidelines for the Use of Animals in Research and the Institutional Animal Care and Use Committee guidelines at the University of Michigan and all field research was conducted with permissions from the appropriate offices in Ethiopia.

### Do males attend differently to high- and low-quality calls based on their own status?

We conducted a playback experiment on 60 adult geladas (20 females, 20 leader males, and 20 bachelor males). To increase our sample size, we included both known and unknown individuals in this experiment. All unknown individuals were identified using morphological features to ensure they were not used in subsequent experiments.

### Playback stimuli

Playback stimuli comprised previously-recorded loud calls obtained during naturally-occurring signaling contests between adult males. Loud calls were recorded using a Sennheiser ME-66 directional microphone and a Marantz PMD 660 digital recorder. Loud call bouts were only used as playback stimuli if they were complete (no calls were missed during the recording) and devoid of background noise and interruptions. We audibly and visually inspected calls using Avisoft SASLab Pro (Avisoft Bioacoustics, Berlin, Germany) acoustic software for acoustic disturbances (e.g., background noise). At the time of the experiment, we had 157 loud call bouts from 50 prime-age males that fit this criteria (e.g., free of background noise).

For geladas, loud call bouts generally consist of a series of two-syllable “ee-yow” calls (2–9 calls per bout). Previously, we found support for the hypothesis that the entire bout (and not just the individual calls within the bout) functions as a quality signal^37^. We selected a total of 14 loud call bouts as playback stimuli: 7 high-quality bouts and 7 low-quality bouts, to construct 10 playback sets, each containing a unique combination of one high- and one low-quality loud call bout from two different males. We determined call quality by comparing calls along several parameters we had previously found to differ with age and status, fundamental frequency and number of calls per bout^37^. To assess these parameters, we conducted a spectrogram analysis in Avisoft with a fast Fourier transformation size of 1024 points (frequency range: 22 kHZ; frequency resolution: 43 Hz time resolution: 2.903 ms; 100% frame). For the 157 loud call bouts, we examined the distribution for both parameters and chose loud call bouts that were at the extremes of these distributions.

### Playback design

We presented each subject with one of the playback sets comprising both a high- and a low-quality loud call bout. Because natural occurrences of loud calls in geladas generally occur when leader males encounter bachelor males, experiments were only conducted when both bachelors and leader males were present on a given day. To simulate a natural loud call contest, the calls were played from the direction of the bachelors (when the subject was a unit individual) or from the direction of the units (when the subject was a bachelor male).

For each trial, we placed a Bose Roommate II portable speaker approximately 25–50 m (M = 28.96 m, SD = 7.95 m) from the subject. The speaker was hidden behind a physical barrier (i.e., tree, rock, or bush), and completely obscured from the subject’s view. All subjects were observed for 15 minutes prior to the start of the playback experiment; and experiments were only conducted if (1) the subject was sitting (e.g., feeding or resting) for at least 2 minutes prior to the start of each call, and (2) the subject was oriented away from the speaker.

The experiment used a within-subjects design, in which subjects heard both a high-quality loud call bout and a low-quality loud call bout in each trial (to simulate a loud call contest between many males). The second call was played 5 minutes after the first call to allow subjects to return to an initial resting state. Subjects generally returned to an initial resting state within 1 minute after hearing the first call. We played each set of calls (n = 10 unique sets) to 6 subjects each: two bachelors, two leaders, and two females. To combat any order effect, we counterbalanced the order in which the high- and low-quality bouts were played across leaders, bachelors, and females. No subject heard any of the calls in the set prior to his or her experimental trial, and each trial was separated by at least 10 days for individuals in the same band.

Prior to all trials, we noted the identity of the subject, the location of the speaker relative to the subject, the subject’s initial state (feeding or resting), the experimental playback set used, and the order of calls heard. During each trial, one observer played the loud calls from a loudspeaker using an MP3 player (Apple ipod touch 3^rd^ generation). A second experimenter with a Kodax PlaySport (Z × 5) HD video camera, positioned herself 5–10 m in front of the subject, with the speaker hidden to the left or right of the subject. All subjects were video-recorded continuously from 15 seconds prior to the first call to 5 minutes after the second call. For each individual, we matched his or her state (feeding or resting) and distance to the speaker between the first and second call – in some cases, moving the speaker to a new hiding spot the appropriate distance away.

For playback trials on unit individuals (leaders and females), we pre-designated two different subjects prior to the start of the trial: a unit male from one unit and a unit female from a different unit at least 40 m away. In such cases, we placed the speaker between the two subjects (from the direction of the bachelors) to ensure their visual trajectories towards the stimuli were not overlapping. Each subject was filmed and scored independently. Experimenters were in contact via two-way radios, and if any of the conditions were not met, we aborted the experiment immediately. We conducted a total of 60 successful playback trials and an additional 22 trials were aborted prior to completion.

### Playback responses

All videos were scored on a computer with a frame-by-frame analysis using Adobe Premier (Adobe Systems, Inc.) by two independent observers. Prior to video analyses, playback videos were cut to contain only the response to one loud call bout within a set. All files were then renamed and randomized such that observers were blind to the identity of the subject (i.e., whether he was a unit male or a bachelor male – it was impossible to hide whether the subject was a female) and the condition (i.e., whether it was a high- or low-quality bout). Reliability for all measurements between the two observers was greater than 95% (M = 97%, SD = 1.2%).

We measured 6 different response variables: (1) duration of time spent looking towards the speaker (*look duration*), (2) duration of time spent moving towards the speaker (*approach duration*), (3) latency of look response (*latency to look*), (4) latency of approach response (*latency to approach*), (5) total distance moved towards the speaker (*approach distance*), and (6) total time to return to initial resting state (*resume activity*).

*Look duration* measured the time a subject spent oriented toward the speaker while stationary. When a subject oriented toward the speaker while moving towards it, we recorded the response as *approach duration*. For both duration responses, we measured the total duration of all responses until the subject returned to their initial state for up to 1 minute after playback onset. Time to return to initial state was assessed once an individual spent at least 15 seconds feeding or resting without orienting towards the speaker. We did not record responses after subjects returned to their initial state as such responses were overly influenced by other individuals within the group (unit or bachelor group). We subtracted any time spent looking or moving towards the direction of the speaker during the 15 seconds prior to the onset of the trial. *Latency to look* and/or *latency to approach* were measured as the time from the onset of the playback stimuli until the onset of the subject’s first look and/or movement towards the speaker. Due to the high mobility of the group during feeding, if a subject did not look or move within the first minute after the onset of the stimulus, we assigned the subject’s latency as 60 seconds. We also recorded *approach distance* for all movement toward the speaker in the first minute after the onset of each playback stimulus.

### Playback analyses

To remove redundancy between response variables, we reduced response variables into latent factors using Factor Analyses (FA^51^) with a varimax rotation with SPSS (v.22.0.0.0). We accepted all factors with eigenvalues greater than 1.0, which produced two factors associated with approach and looking behavior (Table 1).

To assess differences due to call quality and status, we constructed two LMMs, one for each factor score from the FA as the dependent variable with status (leader, bachelor, or female), call quality (high or low), and an interaction between both as predictor variables. In each model, we controlled for call order (fixed effect) and subject (random intercept). To assess whether bachelor’s or leaders differed in their response to high and low-quality calls, we conducted two additional a priori contrast (Table 2). We corrected for multiple testing using a Bonferroni adjusted alpha level of 0.025 (0.05/2).

In addition to the FA, we examined whether males differed in overall response towards high and low-quality calls. We calculated “*overall response time”* for each male by summing the time he spent looking *and* approaching the speaker (*look duration* + *approach duration*). We constructed a third LMM with overall response time as the dependent variable, subject as a random factor, status and call quality as fixed effects and an interaction between status and call quality. Since call order was not a significant factor in any of the previous LMMs, we excluded it from this model.

For these models (and all subsequent models), we determined the statistical significance of the full model by comparing its fit using likelihood tests with that of a null model including only the intercept and the random effect (Table 2). We conducted all model analyses in R v.3.2.0 using the “lmer” function in the lme4 packages v.1.1–11^52^ and contrasts using the lsmeans package v. 2.5–5^53^. We visually inspected each model using a Q-Q plot, histogram of residuals, and scatter plot of fitted versus residual values. Residual values for all models were normally distributed.

### Do males attend differently to high- and low-quality calls based on their own quality?

We assigned each male a *call quality score* by examining 12 acoustic parameters related to frequency (e.g., fundamental frequency) and temporal measures (e.g., call duration). Given our previous results that lower frequency calls are energetically-costly to produce^37^, we established a *call quality score* based on the factor analysis (i.e., Factor 1, *spectral measures*). We focused our analysis on the first calls given within a bout (n = 122) as these calls are the lowest in frequency measures (and presumably the highest quality). We calculated a mean for the *spectral measure* scores to establish a call quality score for each male. Because calls that are lower in spectral measures were higher in quality, we multiplied the call quality score by −1, so that a high *call quality score* represents a high-quality call. We then ran two Spearman’s rank-order correlations, comparing each male’s response time to the high- and low-quality playback stimuli to his own call quality score.

### Are males more likely to join a loud call display when they hear calls of similar quality to their own?

We collected all-occurrence behavioral sampling and recorded 423 loud calls across 291 different loud call displays from 20 males across the study period. For all displays, we recorded the identity of all known males that participated in the display as well as the males present in the group that did not participate. We conducted acoustic analyses on all calls in the same way as described above. Again, we focused our analysis on the first calls given within a bout (n = 122) and established a *call quality score* for each male.

To control for a subject’s “familiarity” with the caller, we used all proximity data between the subject and the caller in a social network analyses^34^. We constructed an undirected, weighted network based on male-male association. In this network, males were represented by nodes and the edge weight was given by an association index. This index was calculated as:

$$\begin{array}{c}Association\,index\,males\,A,B=\underline{\#\,of\,times\,male\,A\,seen\,with\,B}\\ \quad \quad \phantom{\rule{5em}{0ex}}\phantom{\rule{4em}{0ex}}\quad \quad minimum\,\#\,of\,times\,male\,A\,or\,male\,B\,seen\end{array}$$

where the numerator is the total number of times males A and B were seen together in the same group, divided by the minimum number of times we observed either A or B in the same group^34^. The association index ranges from 0 (if two individuals were never seen together) to 1 (if they were always seen together). From this network, we used the Louvain community identification algorithm to assign males to “cliques” within their social network. Males associated into two distinct cliques (N = 15 and N = 21 males respectively) with a modularity coefficient of 0.011. Males were considered to be “familiar” with each other if they were assigned to the same clique, and “not familiar” if assigned to different cliques.

To assess if relative call quality or caller familiarity influenced the likelihood that a male would participate in these vocal displays, we conducted a GLMM with a binomial distribution. For each subject, we examined the dyadic calling relationship with other males. The outcome variable in our model was the likelihood that male A participated in a display, given that male B also displayed. This was modeled as the *count of successes*, the number of times male A and male B displayed together, offset by the *count of failures*, the total number of times male A or B displayed (but not both), given that both males could have displayed (e.g., were both present in the group on that day). We included *relative call quality* and *caller familiarity* in the models as fixed effects. *Relative call quality* was calculated for each dyad by taking the absolute value of the difference between the *call quality scores* of both males. The smaller the difference between the two call quality scores, the closer the males were in *relative call quality*. *Caller familiarity* was established for each dyad from the social network analysis. Males were considered to be “familiar” with each other if they were assigned to the same clique, and “not familiar” if assigned to different cliques. We controlled for the identity of both males by including their identification as random variables in the model. Although the majority of calls were given by leader males, occasionally subordinate follower males engaged in these displays. Because leaders are more likely to display than followers, we controlled for status of both males in the model. We compared the full model to a null model, which included only the intercept and random effects. The social network analysis and GLMM were conducted in R 3.2.4 using igraph^54^ and lme4^52^ packages respectively.

### Data availability

Datasets and R scripts generated and analyzed during the current study are available from the corresponding author on reasonable requests. Playback videos are also available upon request.

## Electronic supplementary material

Table S1
