# Supplementary material for: Evidence for mutual assessment in a wild primate
Source: Sci Rep. 2017 Jun 7;7:2952. doi: 10.1038/s41598-017-02903-w (PMC5462830; doi:10.1038/s41598-017-02903-w)
Supplement: Supplementary file 1 — Table S1 [file 41598_2017_2903_MOESM1_ESM.pdf]

Evidence for mutual assessment in a wild primate

Marcela E. Benítez[1], David J. Pappano[2], Jacinta C. Beehner[3,4], and Thore J. Bergman[4,5].

[1]*Department of Psychology, Language Research Center, Georgia State University, Atlanta, Georgia*

[2]*Department of Ecology and Evolutionary Biology, Princeton University, Princeton, New Jersey*

[3]*Department of Anthropology,* [4]*Department of Psychology, and* [5]*Department of Ecology and Evolutionary Biology, University of Michigan, Ann Arbor, Michigan.*

Table S1. Means and SEM for all response variables

	**Leaders**				**Bachelors**				**Females**			
	**High**		**Low**		**High**		**Low**		**High**		**Low**	
	Mean	*SEM*	*Mean*	*SEM*	*Mean*	*SEM*	*Mean*	*SEM*	*Mean*	*SEM*	*Mean*	*SEM*
Look duration (s)	15.56	2.65	9.40	2.50	17.13	3.11	18.20	3.80	4.83	1.42	3.66	0.83
Look latency (s)	9.68	4.85	15.79	5.86	0.96	0.29	0.73	0.11	15.93	5.84	22.11	6.42
Move duration (s)	4.10	2.03	4.57	2.77	1.61	0.90	11.19	3.65	0.00	0.00	0.00	0.00*
Move latency (s)	44.63	5.40	48.81	5.14	51.79	4.49	34.77	6.41	60.00	0.00	60.00	0.00
Distance moved (m)	6.15	3.10	5.30	3.00	7.15	3.89	19.70	5.62	0.00	0.00	0.00	0.00
Resume activity (s)	20.63	3.91	14.80	4.62	22.26	4.12	31.28	5.41	5.56	1.38	5.34	1.65
Overall response (s)	19.66	3.93	13.97	4.07	18.73	3.57	29.39	5.04	4.83	1.42	3.66	0.83

* Females never moved towards the speaker.
